# Nasopharyngeal Carcinoma in Southeast Asia: Current Landscape and Future Priorities

**DOI:** 10.3389/bjbs.2025.15902

**Published:** 2025-12-12

**Authors:** Suat Ming Chan, Ian C. Paterson, Lee Fah Yap

**Affiliations:** Department of Oral and Craniofacial Sciences and Oral Cancer Research & Coordinating Centre, Faculty of Dentistry, Universiti Malaya, Federal Territory of Kuala Lumpur, Malaysia

**Keywords:** nasopharyngeal carcinoma, NPC, NPC incidence, NPC risk factors, SE Asia

## Abstract

Nasopharyngeal carcinoma (NPC) remains a major public health concern with a geographically skewed distribution. The disease is endemic in East Asia (particularly China), Southeast Asia (SEA) and South-Central Asia. Although China contributes the largest share of global NPC cases, several SEA countries consistently report high incidence rates. Despite this substantial burden, NPC remains a neglected disease across much of the region. This review synthesizes and appraises the available evidence on the epidemiology, incidence trends and disease burden of NPC in SEA. High-incidence hotspots persist in Indonesia, Malaysia, Singapore, Vietnam, the Philippines, and Thailand, with particularly striking rates among indigenous populations of East Malaysia. Late-stage presentation is common and survival outcomes in many SEA countries lag behind those observed in better-resourced endemic regions. Socioeconomic disparities in many SEA communities also amplify exposure to key NPC risk factors. This review outlines key region-specific challenges and identifies priority areas for coordinated health system strengthening. We emphasize the urgent need for regionally tailored strategies to mitigate the growing burden of NPC throughout SEA.

## Introduction

Nasopharyngeal carcinoma (NPC) is a distinct type of the head and neck cancer, arising from the epithelial lining of the nasopharynx. NPC is an enigmatic malignancy that displays marked racial and geographical differences. It is rare in most parts of the world, with an age-standardised incidence rate (ASIR) of 1.3 per 100,000 population for both genders [1]. However, NPC is prevalent in Asia, including East Asia, Southeast Asia (SEA) and South Asia, with ASIRs reaching as high as 20/100,000 in certain parts of southern China and East Malaysia [2, 3].

The pioneering work of the late Professor John Ho at the University of Hong Kong in the early 1970s established NPC as a “Cantonese cancer” due to its exceptionally high prevalence among Cantonese-speaking people in Hong Kong and the Guangdong region of southern China [4]. Over the past five decades, China has reported the highest number of NPC cases worldwide, accounting for more than 50% of the total number globally [1]. With the establishment of national cancer registries, it has become evident that NPC is also prevalent across SEA, including multiethnic nations such as Malaysia, Singapore and Indonesia [5]. It was postulated that NPC susceptibility may trace back to the ancient Bai Yue people of southern China, ancestors to many present-day Southeast Asian populations [6]. Anthropological, genetic, and historical evidence suggest that the elevated NPC risk observed in Cantonese and various Southeast Asian groups likely reflects shared ancestral and genetic backgrounds rather than being confined to a single ethnic group.

Beyond genetic predisposition, the distinctive epidemiological pattern of NPC indicates a multifactorial aetiology. Identification of early-life exposure to preserved food as an important causative factor and the discovery of a strong association with Epstein-Barr virus (EBV) have significantly advanced the understanding of NPC carcinogenesis [7]. Dietary and environmental exposures, including alcohol use, tobacco smoking, and occupational formaldehyde exposure, are now well-recognised risk factors of NPC [8–10]. Non-keratinising undifferentiated carcinoma is the most common type of NPC in the endemic regions, accounting for up to 95% of the cases, with a consistent link to EBV infection [11]. In contrast, keratinising squamous cell carcinoma is usually EBV-negative and found in low-risk countries such as United States and Japan [12, 13].

Reflecting its high national burden, most NPC research to date has originated from China, encompassing studies on prevention, early diagnosis, and therapeutic strategies. In contrast, despite its significant public health impact, NPC remains an under-recognised disease in most SEA countries, with comparatively few epidemiological reports that are often dated. A comprehensive understanding of NPC epidemiology and incidence trends in SEA is therefore crucial for guiding targeted prevention, improving early detection, and optimizing resource allocation for cancer control. This review synthesizes current literature and publicly available data to provide an overview of NPC in SEA, highlighting the need for a renewed focus to capture current disease patterns and inform management strategies for NPC patients in the region.

## Search Strategy

The PubMed database and Google search were used to identify eligible articles and publicly available information. The following keywords were applied: “nasopharyngeal carcinoma AND Southeast Asia,” “nasopharyngeal carcinoma AND Asia,” “nasopharyngeal carcinoma AND [name of each country in SEA],” and “cancer registry AND [name of each country in SEA].” No restrictions were imposed on publication date. Initial search results were screened for relevance, and non-English articles were excluded. Only peer-reviewed articles were included. Full texts were retrieved and reviewed to confirm eligibility prior to inclusion in this review.

## Regional Burden of NPC

**FIGURE 1:**
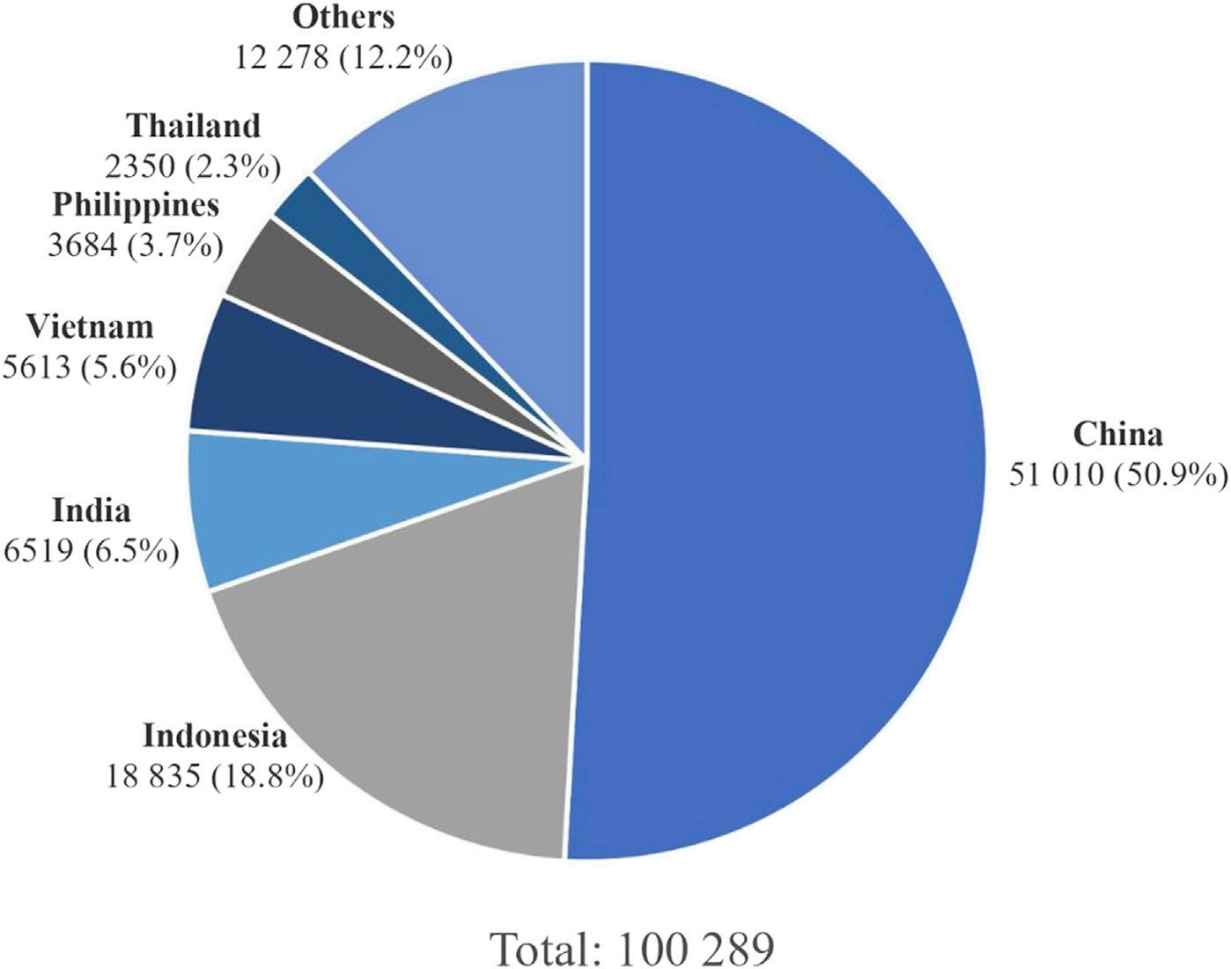
Number of new NPC cases in 2022 in Asia [1].

According to the Global Cancer Observatory (GLOBOCAN) data, an estimated 120,434 new NPC cases and 73,482 NPC-related deaths were reported worldwide in 2022. Approximately 83.3% of cases (100,298 cases) and 83.6% of deaths (61,442 deaths) occurred in Asia, with the highest number of incidences in East Asia (52.5%; 52,610 cases), followed by Southeast Asia (35.8%; 35,889 cases) and South-Central Asia (9.2%; 9,248 cases) [1]. China bears the greatest burden of NPC in East Asia, contributing to over half of all cases worldwide (51,010 cases; 50.9%) and this can be partly explained by the large population base of China (Figure 1). The lowest incidence was in Oceania, with 262 cases reported (Table 1). The highest ASIR of 7.5 was observed in the Maldives, an archipelagic nation in South Asia. However, according to the Human Development Index (HDI) in 2012, Maldives had the lowest ASIR of NPC (0 per 100,000 people) in Asia [5]. Since only 36 NPC cases were reported in Maldives according to GLOBOCAN 2022, the high ASIR was likely attributable to the small population size of the country. Notably, nine of the ten countries with the highest ASIRs are located in SEA, with Brunei, Indonesia, Malaysia, and Singapore comprising four of the top five (Table 2). It is noteworthy that Indonesia accounted for the second highest number of NPC cases (18,835; 18.8%) and recorded the third highest ASIR of 6.1, underscoring NPC as a major public health problem in the country (Figure 1; Table 2).

**TABLE 1:** Numbers of incidence and mortality of NPC in both sexes (GLOBOCAN 2022).

Continent	New cases (number)	Percentage of new cases (%)	Death cases (number)	Percentage of death cases (%)
Asia	100,298	83.3	61,442	83.6
Africa	10,762	8.9	7,029	9.6
Europe	4,513	3.7	2,563	3.5
North America	2,380	2.0	1,262	1.7
Latin America & Caribbean	2,219	1.8	1,041	1.4
Oceania	262	0.22	145	0.2

**TABLE 2:** Worldwide ASIRs of NPC in both sexes (GLOBOCAN 2022).

Ranking	Population	ASIR
1	Maldives	7.5
2	Brunei	6.4
3	Indonesia	6.1
4	Malaysia	5.9
5	Singapore	5.4
6	Timor-Leste	5.0
7	Viet Nam	4.7
8	Lao PDR	3.8
9	Myanmar	3.5
10	Philippines	3.5
11	China	2.4
12	Thailand	2.2
13	Cambodia	2.1
14	Bhutan	1.7
15	Yemen	1.5

Gender disparity is a well-recognised feature of NPC, with incidence rates in men typically two to three times higher than in women [14]. This epidemiological pattern is consistently observed across SEA. Among SEA countries, Indonesian and Malaysian men exhibited the highest ASIRs of NPC, at 9.6 and 8.9, respectively, as compared to China men with ASIR 3.4 (Table 3). This difference might be attributed to gender-specific behavioural and environmental exposures, such as higher rates of tobacco use, alcohol consumption, and occupational exposure to carcinogens among men [15, 16]. Additionally, potential biological influences, including the protective effect of sex hormones and inherited susceptibility loci on the X-chromosome, cannot be ruled out [17, 18]. Although overall survival outcomes do not appear to differ significantly between sexes, the greater tendency for men to present with advanced disease underscores the need for targeted prevention and early detection strategies.

**TABLE 3:** Gender-specific ASIRs of NPC in China and SEA countries (GLOBOCAN 2022).

Country	New cases	ASIR per 100,000 males	ASIR per 100,000 females
China	51,010	3.4	1.3
Indonesia	18,835	9.6	2.8
Vietnam	5,613	7.1	2.5
Philippines	3,684	5.2	2.1
Thailand	2,350	3.3	1.2
Malaysia	2,144	8.9	2.8
Myanmar	2097	5.1	2.1
Singapore	540	8.0	2.7
Cambodia	310	3.2	1.0
Laos	236	5.0	2.7
Timor-Leste	48	7.2	2.7
Brunei	32	8.7	4.0

## Country-Specific Incidence and Trends

NPC exhibits marked disparities in incidence and outcomes across ethnic groups, particularly in SEA, where many multiethnic nations comprise both Chinese and non-Chinese populations. No peer-reviewed publications on NPC were found from Myanmar, Cambodia, Laos, or Timor-Leste. Although Brunei recorded the second highest ASIR (6.4 per 100,000) globally, only 32 cases were reported (GLOBOCAN 2022), suggesting that its small population may have inflated the rate. Therefore, these countries are not discussed further.

### Indonesia

While the Indonesian government is currently developing a national population‐based cancer registry system, existing epidemiological data on NPC in Indonesia are derived exclusively from hospital-based medical record registries, and therefore may not represent the disease landscape in the country. An early report from 2012 described NPC as the fourth most common malignancy in Indonesia [19], and this was supported by a recently published imPACT Review coordinated by the Ministry of Health Indonesia [20]. Indonesia comprises more than one thousand ethnic groups across 38 provinces [21] and published data were predominantly originated from hospitals located at Western Indonesia, including Java and Sumatra. Studies conducted at Dr. Cipto Mangunkusumo National Hospital Jakarta showed that between 1996 and 2011, over 60% of NPC patients were of Javanese and Sundanese origin, whereas Chinese patients accounted for 10% or fewer of cases [19, 22]. These findings suggest that NPC incidence in Indonesia did not correspond closely to the demographic distribution of Chinese descendants, despite their large population in Jakarta and surrounding areas. Nevertheless, comprehensive national-level data are needed to draw definitive conclusions.

Late presentation remains a major challenge in NPC management, particularly in Indonesia. Reports from Yogyakarta (Dr. Sardjito General Hospital), Jakarta (Dr. Cipto Mangunkusumo National Hospital) and East Java (Dr. Soetomo General Hospital) indicated that 51%–70.5% of NPC patients presented with Stage IV disease [22–24]. Strikingly, findings from Dr. Kariadi General Hospital in Central Java indicated that 31.6% of patients diagnosed at an advanced stage presented with distant metastases (Stage IVC), highlighting the burden of late presentation [25]. NPC primarily affects adults of productive age, with incidence peaking between 40 and 59 [26]. Although a bimodal age distribution has been described in North Africa, showing a modest rise in incidence among those aged 10 to 25 [27], NPC is rarely seen among adolescents in Asia. Notably, in Jakarta, 20% of NPC patients (1,121 cases diagnosed between 1996 and 2005) were younger than 31 years, without evidence of a bimodal age distribution [19]. A subsequent study at the same hospital identified 49 NPC patients under 31 years of age, of whom 79.6% presented with Stage IV disease and 14% had distant metastases [28]. Similar trends were observed in an independent study from Dr. Zainoel Abidin General Hospital, Banda Aceh (2014–2019), where the mean age at diagnosis was 17.2 years, and most patients were diagnosed during late adolescence and early adulthood (53% aged 17–25 years; 47% aged 12–16 years) [29].

The prognosis for NPC patients in Indonesia is relatively poor. An early study in Yogyakarta reported a median survival of 21 months among 78 cases diagnosed between 2008 and 2011 [24]. A subsequent analysis involving a larger cohort of 759 patients diagnosed between 2007 and 2016 at the same hospital found a median observed survival of 31.08 months [30]. When extrapolated to a five-year period, the overall survival rate reached only 35%. Similar findings were reported in Jakarta, where a study showed a two-year overall survival rate of 39%–71% among young, non-metastatic patients [28]. Collectively, these results indicate that NPC survival outcomes in Indonesia were lower than those reported from neighbouring endemic Asian countries, where treatment with induction chemotherapy (IC) and radiotherapy yielded survival rates of 54.7%–83.2% during that period [31, 32]. Encouragingly, a more recent study from Central Java involving 50 patients diagnosed in 2018 reported a three-year overall survival rate of 60.8% following chemoradiation therapy [25]. However, no statistically significant difference in overall survival was observed between early- and advanced-stage patients in this study, a finding that contrasts with earlier studies from Yogyakarta and other Asian populations [30, 33, 34].

### Malaysia

In Malaysia, NPC has ranked among the top ten most common cancers since the first Malaysian National Cancer Registry Report 2002 was published (https://nci.moh.gov.my/index.php/ms/main-menu-2/laporan), indicating that NPC is a major national public health issue. Malaysia is a multiethnic country comprising three main racial groups, namely Malay, Chinese and Indian, each with distinct social and cultural backgrounds. The genetic contribution to the pathogenesis of NPC in Malaysia is evident. Both Chinese males and females consistently show higher ASIRs than their Malay and Indian counterparts (Table 4). According to the Malaysian National Cancer Registry Report 2017–2021, the lifetime risks of developing NPC are 1 in 101 for Chinese, 1 in 344 for Malay and 1 in 1,233 for Indian [40]. Although a declining trend in ASIR has been observed among Chinese people over the past two decades, Malaysian Chinese males still demonstrate a higher ASIR (8.6 per 100,000 people) compared to males in China (3.4 per 100,000 people). Interestingly, an early report identified variations among Chinese subgroups, with Cantonese males showing the highest ASIR of 17.3, followed by the Hakka (Khek), while Hokkien and Teochew subgroups reported lower rates [35]. Previous studies have shown that Chinese migrants from southern China/Hong Kong to the United States retained a comparably high NPC incidence, but the risk decreased progressively across successive generation born in the United States [41]. The first generation of Malaysia Chinese was born in the 1900s, and even after more than a century of local residence, the persistently elevated ASIRs among this population suggest that environmental exposures may play an equally important role as genetic predisposition in determining NPC risk in Malaysia.

**TABLE 4:** Ethnic-specific ASIRs of NPC in Malaysia.

Study period	ASIR (males)			ASIR (females)			Population	References
	Chinese	Malay	Indian	Chinese	Malay	Indian		
1968–1972	17.3	2.5	1.1	7.3	0.3	NA	State of Selangor	[35]
1968–1977	16.2–16.8	2.8–1.9	1.2–0.8	7.0–7.3	0.3–1.0	NA	National	[36]
1988	10.7	1.0	0.3	3.9	0.3	0.2	National	[37]
2002	23.0	5.6	4.0	10.3	2.3	1.7	National	Malaysia cancer registry report 2022
2003–2005	17.0	4.2	2.0	6.6	1.6	1.0	National	Malaysia cancer registry report 2003–2005
2007–2011	11.0	3.3	1.1	3.5	1.3	0.6	National	Malaysia cancer registry report 2007–2011
2012–2016	8.6	2.7	0.6	2.9	1.1	0.4	National	Malaysia cancer registry report 2012–2016
2012–2017	7.0–1.3	3.5–1.4	NA	1.2–0	1.5–0.7	NA	State of Pahang	[38]
2015–2019	2.13	0.35	NA	0.65	0.15	NA	State of Sabah	[39]

NA, not available.

Regional disparities significantly influence the burden of NPC in Malaysia. Notably, the two states located on the island of Borneo, Sarawak and Sabah, recorded the highest ASIRs of NPC among all 13 Malaysian states [42]. The populations of Sarawak and Sabah comprise a multitude of ethnic groups, with indigenous peoples accounting for approximately 60%–70% of the total population. A landmark study published in 2004 reported that between 1996 and 1998, the Bidayuh, a native group in Sarawak, exhibited the highest ASIRs globally, reaching 31.5 in males and 11.8 in females [3]. The elevated incidence among the Bidayuh has persisted over time; a more recent analysis of 3643 NPC cases in Sarawak (1996–2015) found ASIRs of 27.8 in males and 11.6 in females [43]. These data are comparable to those reported in high-incidence regions of southern China, such as Zhongshan and Zhuhai regions, where ASIRs were approximately 25 for males and 9 for females [44]. In neighbouring Sabah, elevated NPC incidence rate has similarly been observed among indigenous populations, particularly the Dusun and Kadazan groups, although their ASIRs were lower than those reported in Bidayuh [39, 45, 46].

Consistent with global trends, Malaysia demonstrates a predominant pattern of late-stage NPC diagnosis. A multi-institutional prospective study conducted between July 2007 and February 2008 reported that 47% of new NPC cases were diagnosed at Stage IV [47]. Sadly, this challenge persisted over time. Even after more than a decade, 46.7% of NPC cases remained at Stage IV at initial presentation, according to the Malaysian National Cancer Registry Report 2017–2021. Although juvenile NPC is less common in Malaysia than in Indonesia, it has been documented among the Kadazan, the indigenous people of Sabah, in several early studies [45, 46, 48]. The first report on NPC incidence in Sabah, published only in 2021, did not provide age-specific demographic data [39]. Therefore, further study is warranted to evaluate the prevalence of juvenile NPC in Malaysia.

Existing publications on NPC survival outcomes in Malaysia are relatively dated. The unavailability of intensity-modulated radiotherapy (IMRT) facilities in public hospitals (Penang General Hospital and Hospital Kuala Lumpur) and academic medical centres (University Malaya Medical Centre and Hospital Universiti Sains Malaysia) was associated with unsatisfactorily low 5-year overall survival (OS) of 33.3%–38% for patients treated between 1998 and 2007 [49, 50]. Subsequent studies involving patients treated between 2002 and 2008 reported an improvement in OS to 51.6%–58.6%, largely attributed to the increased adoption of concurrent chemoradiotherapy (CCRT) [51, 52]. Nevertheless, these outcomes remained inferior to those achieved with IMRT-based treatment, where 5-year OS rates exceeding 80% have been reported in developed centres [53]. Consistent with this, a private medical centre in Malaysia, where IMRT is the standard of care, reported a respectable higher 5-year OS of 73% [54]. IMRT-based treatment has only been implemented in Malaysian public hospitals since 2011 [55], and outcome analyses from major cancer centres are eagerly awaited.

### Singapore

Singapore is a compact nation characterized by its highly urbanized setting. The Singapore Cancer Registry, established in 1967, monitors population-based cancer trends and patterns across the country. In the most recent Singapore Cancer Registry Report 2018–2022, NPC is not listed among the ten most common cancers overall [56]. However, it remains clinically relevant; NPC ranks 10th in cancer-related mortality among men, and is the third most common cancer in males aged 30–49 years. In the younger population (0–29 years), NPC also appeared as the 10th most common cancer, with 22 cases recorded during the reporting period. As young-onset NPC is rare, particularly in urban settings, it would be valuable to investigate whether its occurrence in Singapore reflects underlying genetic susceptibility or lifestyle changing, such as increasing alcohol consumption.

The ethnic makeup of Singapore closely mirrors that of Malaysia, comprising three main groups, namely Chinese, Malay and Indian. Unlike Malaysia where Malay accounts for more than 70% of the population, Singapore is predominantly Chinese (approximately 74%). Consistent with demographic patterns of Chinese ancestry, the ASIR of NPC is highest among Chinese, intermediate among Malays, and lowest among Indians [57]. Earlier analyses have also reported variation within Chinese subgroups; major dialect groups (Hokkien, Teochew, Cantonese, Hainanese, Hakka) all showed elevated risk, with Cantonese people exhibiting particularly high susceptibility [58]. A secular trend analysis covering 1973–1997 reported a significant decline in NPC incidence, especially among cohorts born after 1958 [59], suggesting environmental and lifestyle changes might have contributed to the reduction of NPC over time.

Across many SEA countries, close to 50% of NPC patients present with Stage IV disease. In contrast, the proportion appears lower in Singapore. At the National University Hospital (NUH) Singapore, 35.5% of patients treated between 2002 and 2012 were diagnosed at Stage IV [60]. Similar findings have been reported at the National Cancer Centre Singapore (NCCS), where 30% of patients treated between 2002 and 2005 and 32.6% treated between 2017 and 2023 presented with Stage IV disease [61, 62]. Notably, the latter study reported no cases of Stage IVc at diagnosis. A dedicated epidemiological investigation would help determine whether this represents a genuine downward trend and whether improved awareness was driven by higher education levels. When comparing ethnic groups, non-Chinese patients (Malay, Indian, Caucasian, Kenyan and others) were more likely to present with advanced nodal disease, although their OS did not differ significantly from Chinese patients [60].

Singapore is classified as a country with a very high HDI. Early-stage disease (stage I/II) is treated with radiotherapy (RT) alone or CCRT, while stage III/IV disease is treated with either CCRT or IC followed by CCRT, with or without adjuvant therapy [61]. IMRT has been used at NCCS since 2002, and a cohort of 195 IMRT-treated patients (2002–2005) demonstrated an impressive 3-year overall survival of 94.3% [62], comparable to other international centres. IMRT also became the standard radiotherapy modality at the NUH in 2006 [60]. Taken together, Singapore demonstrates some of the strongest NPC management outcomes in SEA, largely attributable to its high socioeconomic development and strong healthcare infrastructure, resulting in superior survival outcomes for NPC patients.

### Vietnam

Published data on the incidence of NPC in Vietnam are quite limited but the epidemiological pattern of NPC in Vietnam appears to mirror that of other SEA countries. The first cancer registry in Vietnam was established in 1988 in Hanoi followed by a registry in Ho Chi Minh City (HCMC) in 1990. Other regional registries have subsequently been set up, but most available data come from Hanoi and HCMC, which have been reported to generate the best quality data in the country [63]. Data from these registries is used by The GLOBOCAN to model national cancer statistics and the most recent estimate indicates that NPC is the 9^th^ most common cancer in Vietnam and the 7^th^ highest cause of cancer mortality [1]. In Vietnam, local data indicate that NPC was the 5^th^ most common cancer in males between 2000 and 2018 [63].

Data from a number of studies demonstrates that NPC in Vietnam exhibits similar characteristics as those in other SEA countries, including age, gender and histological subtypes [64]. As in other SEA countries, patients appear to present with late-stage disease, but the data are limited. In a small single-centre study in Hanoi of 31 patients, 67.74% of patients presented with late-stage disease (stage III-IV), where 38.71% accompanied by tissue invasion and distant metastasis [65]. In a more comprehensive study of 8974 cases of head and neck cancers, NPC contributed the highest proportion of stage IV cases (14.53%) among these tumours [64], supporting the overall premise that NPC is often diagnosed at late stages. There is some evidence to indicate a geographical variation within Vietnam. Data from the Hanoi and HCMC cancer registries in 2000 indicated that NPC was more frequently observed in Hanoi (ASIR of 10.7 in males, 5.1 in females) than in HCMC (ASIR of 5.1 in males, 1.5 in females) [66], but this requires confirmation.

Published data on prognosis and survival outcomes in Vietnam remain limited. In a recent cohort of patients with advanced-stage (III/IVA) undifferentiated NPC treated at the Vietnam National Cancer Hospital (2021–2024), 96.6% received IC followed by CCRT. IMRT was administered to 70.7% of patients, and 77.5% achieved disease-free survival beyond 12 months [67]. These findings highlight the need for more comprehensive epidemiological and clinical studies to better characterise the NPC landscape in Vietnam.

### Philippines

To date, only one study has assessed the incidence of NPC in Philippines. The country has two recognised population-based cancer registries, namely the Philippine Cancer Society-Manila Cancer Registry (PCS-MCR) and the Department of Health-Rizal Cancer Registry (DOH-RCR), which cover Metropolitan Manila and the nearby Rizal Province. In collaboration with the World Health Organisation International Agency for Research on Cancer (WHO IARC), these registries have made significant contributions to cancer data collection in the Philippines. Between 1980 and 2007, data from these registries consistently ranked NPC among the top 10 cancers in Filipino males [68]. However, these sources do not comprehensively represent all regions of the Philippines. In recognition of this limitation, a multicentre study was conducted across four major oncology centres in different cities, University of Santo Tomas (Manila), Makati Medical Centre (Makati), Philippine Oncology Centre Corporation (Quezon), and Cardinal Santos Memorial Medical Centre (San Juan), to collect demographic data on NPC patients [69]. Although the sample size was small (49 NPC patients), the study provided insights not captured by the IARC reports. Particularly, it showed that approximately one-third of cases originated from the northern and central Luzon provinces (Manila is at the southern Luzon), with incidence increasing with age and peaking at 50–59 years. Consistent with trends in other SEA countries, 44.9% of cases were diagnosed at Stage IV [69]. This study also highlighted that NPC incidence in the Philippines may have been underreported.

### Thailand

Thailand currently has 16 population-based regional cancer registries that collectively provide national coverage across different regions. In collaboration with these registries, National Cancer Institute of Thailand publishes the *Cancer in Thailand* monograph, a series of comprehensive statistical reports on cancer incidence and epidemiology. Although NPC is not listed among the ten most common in Thailand in the latest monograph 2019–2021 [70], it ranks 7^th^ among cancers in men according to the 2023 hospital-based cancer registry [71]. While the ASIR of NPC in Thailand (2.2 per 100,000 people) is considerably lower than in neighbouring SEA countries, the country still ranks 6^th^ worldwide in NPC incidence [1]. These data underscore that NPC remains an important cancer in Thailand. The comparable ASIRs across all regions indicate no significant geographical disparity within the country [70].

NPC is well recognised to be predominantly of the non-keratinising subtype in endemic regions [72]. An analysis of 1990–2014 data from four Thai registries (Chiang Mai, Khon Kaen, Songkhla, and Lampang) provided additional insight [73]. Non-keratinising subtype constituted 66%–79% of cases in Chiang Mai, Khon Kaen, and Lampang. In contrast, in Songkhla (located at the southern Thailand), the keratinising subtype accounted for approximately 60% of cases, and the authors attributed to historically high smoking prevalence in the region. Nonetheless, the incidence of keratinising NPC has significantly declined over time and non-keratinising NPC now represents the predominant subtype in Thailand [74, 75].

Although comprehensive, the *Cancer in Thailand* monograph does not include information on disease staging. Evidence from independent studies, however, indicates that late-stage presentation is also common in Thailand [74, 75]. Between 2008 and 2020, 43%–45% of NPC patients treated in hospitals in Bangkok and Songkla presented with Stage IV disease. A multicentre study involving four major Thai cancer centres (Ramathibodi and Siriraj Hospitals at Mahidol University, King Chulalongkorn Memorial Hospital and Songklanagarind Hospital) reported that CCRT was the predominant treatment modality from 2008 to 2020, with a 5-year OS rate of 62% [75]. The widespread adoption of IMRT and volumetric modulated arc therapy (VMAT), supported by national health coverage since 2007, has contributed to significant improvements in survival and reductions in treatment-related toxicities. Patients treated with IMRT/VMAT achieved a 5-year OS of 76%, compared with 51% for 2D techniques and 58% for 3D conformal radiotherapy.

## Association of Lifestyle and Environmental Factors With NPC in SEA

NPC exhibits a remarkable predilection for certain populations and geographical regions, even within the same country. Although genetic predisposition is a well-recognised risk factor, these disparities are likely influenced by a complex interplay of social and environmental factors that extend beyond genetic or ancestral origins. An analysis of NPC incidence in SEA indicated that the disease is more prevalent in countries with moderate levels of development than in those that are highly or very highly developed [76]. Socioeconomic status has been associated with NPC incidence, potentially due to greater exposure to risk factors among populations with low socioeconomic status [77, 78]. A multicentre study showed that poorer residential conditions, including housing type, cooking fuel type, ventilation condition of house, source of drinking water and household air pollution were linked to an increased risk of NPC [79]. These residence characteristics are particularly relevant in rural areas of developing countries, where infrastructure is under-developed. Furthermore, individuals with low socioeconomic status are more likely to present with advanced-stage disease and subsequently poorer survival outcomes [80]. In support of this, lower educational attainment was found to be positively associated with NPC incidence in Thailand and reduced patient survival in Indonesia [30, 73].

It is now widely recognised that lifestyle factors, such as smoking, alcohol consumption and dietary habits, particularly the intake of salt-preserved foods, are associated with an increased risk of NPC [81]. It is noteworthy that the IARC has classified preserved food containing nitrosamine, such as Chinese-style salted fish, as a carcinogen for NPC [82]. Numerous reports from SEA provide evidence consistent with this notion. Early studies reported that a history of consuming salted fish intake during childhood which continued into adolescence, was a significant risk factor for NPC among Malaysian Chinese populations [83, 84]. Subsequent case-control studies further demonstrated that high consumption of salt-cured food, red meat, pork/beef liver and alcohol combined with a low intake of fruits and vegetables, was associated with an elevated NPC risk among Malaysian patients [85, 86]. Similar observations were reported in Indonesia, where salted fish consumption was identified in 29.9% of 167 NPC patients [22]. Likewise, in Singapore, younger Chinese NPC (<45 years) who frequently consumed salted food were found to have a higher risk of developing NPC [87]. A subsequent study in 2017 further confirmed that frequent consumption (once a week or more) of salted vegetables, but not salted fish, was a significant risk factor for NPC among Singapore patients [88]. In Thailand, fermented vegetables (*pak-kad-dorng*), rather than salted fish, were linked to an increased risk of NPC [89]. A similar finding was reported in the Philippines, where salted fish consumption was not associated with NPC risk [90]. These differences have been speculated to arise from variations in salted fish preparation methods, differences in average consumption levels, or the age at exposure.

Dietary patterns may explain the high NPC incidences observed among the Bidayuh population in Malaysia, whose traditional ethnic native food commonly includes preserved pork and fish [91]. Notably, the Bidayuh people also exhibits the highest incidence of gastric cancer among men in Sarawak, a malignancy strongly linked to high-salt diets [92]. It is important to note that these ethnic native foods are typically homemade, without standardised quality control or regulation of preservative levels. A similar phenomenon is thought to occur in Indonesia; in addition to the widespread consumption of dried salted fish, carcinogenic substances such as formalin and polyaromatic chemical dyes is rather common in food supply in local markets and small-scale factories [93, 94].

The association between tobacco smoking and NPC risk is established [95]. In the Philippines, long-term smoking (>30 years) increased 7.2-fold risk of developing NPC [90]. Consistent findings were reported in Thailand and Singapore, where both current and former smokers had approximately twice the risk of NPC compared to never-smokers [88, 89, 96]. In addition to cigarette smoking, waterpipe smoking represents another traditional form of tobacco exposure. Among men, the prevalence of waterpipe use is highest in Vietnam, followed by China and Malaysia [97]. A study involving 20,144 Vietnamese men revealed a significant positive association between exclusive waterpipe smoking and NPC, suggesting that waterpipe smoking is likely to confer a greater carcinogenic risk than cigarettes [98].

The potential exposures to various occupational factors, such as wood dust, textiles, organic solvent or tar, are also linked with an increased risk for NPC [99]. Supporting this, IARC has classified both wood dust and formaldehyde as carcinogenic agents for NPC [82]. In Malaysia, Chinese individuals working in industrial sectors and residing in socioeconomically disadvantaged neighbourhoods were found to have higher incidence of NPC [36]. Similarly, occupational exposure to wood dust was identified as a significant risk factor among Thai NPC patients [96], while studies in Malaysian Chinese populations found that exposure to smoke, dust (including wood dust), and industrial heat were associated with increased NPC risk [84, 100]. However, no association between formaldehyde exposure and NPC was observed in the Malaysian study [100], contradicting earlier reports [100, 101]. Interestingly, an early study from Philippines demonstrated that both the latency period and younger age at first exposure to formaldehyde, dust, or exhaust fumes were strongly associated with elevated NPC risk, with up to 4-fold among those exposed at a young age [90]. It has been speculated that occupational hazards such as wood dust and solvents, may partly explain the high incidence of NPC among the Bidayuh people in Malaysia, as the timber and rubber industries are major economic sectors in Sarawak [91]. Nevertheless, the existing literature on risk factors for NPC in Malaysia remains dated, highlighting the need for contemporary, population-specific studies to better characterize these associations.

## Systemic Challenges in NPC Management in SEA

In addition to individual determinants, systemic challenges significantly complicate the management of NPC in SEA. Although these issues are not exclusive to the region, they tend to be more pronounced in SEA due to infrastructural and socioeconomic disparities.

### Low Public Awareness and Health Literacy

NPC frequently presents at an advanced stage, largely due to its hidden anatomical location and the non-specific nature of early symptoms (such as nasal congestion or discharge), which are often overlooked or misattributed to benign conditions [102]. Most patients seek medical attention only when visible neck lumps appear, making this the most common presenting symptom [103]. This delay is particularly relevant for patients in rural areas, where educational levels and socioeconomic status are generally low. A Malaysian study found that 41.5% of rural respondents had limited health literacy [104]. In Sarawak Malaysia, a study involving 216 patients reported a mean delay of 176 days from symptom onset to seeking professional attention [105]. The primary reasons identified were lack of awareness of NPC and its seriousness (72%), absence of pain (30%) and initial reliance on traditional treatment (24%). A similar pattern was observed in Indonesia, where a majority of the patients experienced unilateral ear problem (the earliest sign of NPC) several months before presenting with cervical lymph node enlargement (a sign of late stage NPC) [19]. In the Philippines, the average duration of symptoms before first consultation was 18.3 months, with 78% (28 of 36) of patients had symptoms more than 3 months at presentation [106]. Beyond limited awareness, cultural reliance on traditional medicine further delays diagnosis and treatment. Some patients abandon standard treatment mid-course in favour of alternative medicine, which are often unregulated and lack scientific validation [91]. Denial of diagnosis also contributes to treatment delays.

### Delay in Diagnosis by Healthcare Providers

Early detection of NPC is often hindered by its symptom overlap with common upper respiratory infections, leading to misdiagnosis by general practitioners (GPs) and thereby a delay in referral. A study in Yogyakarta Indonesia revealed that knowledge on NPC of GPs working in the Primary Healthcare Centres was insufficient, with many of them were unaware of NPC prevalence in their region [107]. This knowledge gap stems from insufficient training during medical education and limited knowledge in clinical practice. Similarly in Kuala Lumpur Malaysia, an early study revealed delayed diagnosis was attributed to poor awareness among primary healthcare workers, with a median diagnostic delay of 127 days [108]. Furthermore, NPC symptoms such as blurred vision and diplopia may prompt patients to consult ophthalmologists, underscoring the need for broader awareness among various medical specialties [106]. Enhancing education and training for regional healthcare providers is a critical first step for timely diagnosis and intervention.

### Prolonged Overall Treatment Time

Treatment delays also arise from prolonged overall treatment time (OTT) in SEA countries. Interruptions in radiotherapy causing a prolonged OTT have been reported to be detrimental for local disease control and survival outcomes of NPC [109]. An early study in Kuala Lumpur Malaysia showed that only 72.2% of patients completed their treatment within the recommended OTT [52]. This issue was further well-documented in a study in Yogyakarta Indonesia involving 68 patients between 2011 and 2012 [110]. The results showed that limited radiotherapy units led to treatment delay of 3–4 months and extended OTT by 10–12 days, significantly affecting treatment outcomes. In part this issue is often caused by equipment downtime due to poor maintenance or aging infrastructure. While urban centres may have addressed these challenges, they likely persist in rural areas across SEA.

### Limited Access to Healthcare Facilities

Access to healthcare facilities remains a major barrier to the timely diagnosis and treatment of NPC in SEA, particularly in rural and remote regions [73, 91]. Geographic isolation, inadequate infrastructure, and limited transportation options significantly hinder patients from reaching appropriate medical services. In many rural areas, hospitals or oncology centres are located far from communities, requiring patients to travel over long distances. For example, in Sarawak Malaysia, patients from interior regions must rely on logging roads or rivers to reach tertiary hospitals, creating substantial delays and discouraging treatment-seeking behaviour [111]. Similar challenges are reported in Indonesia, where rural patients frequently struggling with the absence of reliable public transport and long travel times to urban medical centres [112]. Beyond the direct costs of treatment, the urban-centric distribution of healthcare providers compels rural patients to incur additional costs for travel, accommodation, and caregiving. These financial and logistical burdens often lead to delays in seeking care or, in some cases, abandoning treatment altogether. Although this issue has not been specifically documented in the literature as directly influencing NPC outcomes in SEA, it has been implied in a study from Thailand [73]. Northern Thailand encapsulates a “hill tribe” population; incidentally, registries from Chiang Mai and Lampang (north) reported poorer survival outcomes compared with those from Khon Kaen (northeast) and Songkhla (south). Given that limited access to healthcare is a well-recognised challenge across many developing countries, it is highly plausible that NPC patients in SEA experience similar barriers.

## Future Directions

A recent analysis utilizing data from the Global Burden of Disease Study 2019 (https://ghdx.healthdata.org/gbd-2019) reported that the incidence number, ASIR, prevalence number, and age-standardised prevalence rate (ASPR) of NPC in China showed an increasing trend from 1990 to 2019, and are projected to continue rising through 2035 [8, 113]. A similar pattern is likely occurring in SEA, because Malaysia is projected to experience a 63.7% increase in incidence rates between 2019 and 2035 [8]. At present, cancer registry systems across SEA remain fragmented and insufficiently developed; most registries likely do not capture cases from the entire country, resulting in under-reporting of NPC. Comprehensive, nationwide cancer registries are indispensable for understanding the true epidemiological burden of the disease. Further, in many SEA countries, NPC patient registration is not digital, leading to inconsistent or unavailable follow-up information. The need for robust, digitised patient registration systems, such as electronic health records (EHRs), cannot be overstated, as they form the foundation for accurate and effective surveillance, treatment monitoring, and outcome assessment.

NPC patients are generally diagnosed at a late stage and the associated treatment imposes significant socioeconomic strain on patients and their families. The treatment strategy profoundly influences survival outcomes. Although IMRT-based radiotherapy is the global standard of care, access remains limited in parts of SEA. Strengthening health system capacity and optimisation of service delivery would broaden access to guideline-recommended treatments and likely improve survival outcomes. In addition, immunotherapy is emerging as an important therapeutic option for many types of cancer, including NPC. Toripalimab and Penpulimab are currently two United States Food and Drug Administration (FDA)-approved immunotherapy drugs for recurrent or metastatic NPC [114]. However, in many SEA countries, access to these drugs is limited to those who can afford it, preventing most patients from receiving optimal care. Improving affordability and access to novel cancer therapeutics is therefore an urgent priority in SEA.

Delays in NPC diagnosis, both patient- and provider-related, must also be addressed. At the patient level, awareness initiatives to promote healthy lifestyle and early symptoms recognition are essential. Exposure to salt-preserved food or environmental carcinogens during childhood increases NPC risk and may partly explain the high burden of juvenile NPC observed in Indonesia and possibly Sabah, Malaysia. Public education campaigns targeting rural populations with lower socioeconomic status are needed to encourage modifications in traditional diets and environmental exposure. Meanwhile, rapid economic and social change in urban areas may shift risk patterns; although consumption of salted foods may decline, alcohol intake and smoking rates may rise. This is consistent with recent data from China showing increased NPC incidence among younger and middle-aged individuals [113]. National or regional NPC awareness programmes would be timely to promote symptom recognition, healthy lifestyles, and early medical seeking behaviour.

At the health service level, particularly in high-risk regions, training programmes for primary healthcare providers, led by otorhinolaryngology or oncology specialists, is crucial to improve recognition of early, often non-specific signs and symptoms of NPC. This approach has shown success in Indonesia, where knowledge among primary healthcare workers remained improved 1.5 years after NPC-focused training [115]. It is also essential to reduce complex referral processes that may prolong the interval between first presentation and diagnosis. Collectively, improving early detection requires simultaneous efforts by both patients and healthcare providers.

Enhancing access to healthcare for underserved communities is another critical step. For example, some pilot initiatives in Malaysia, Thailand and Indonesia, such as subsidised transport programmes and mobile outreach clinics, have shown promise in improving access to care. To bridge systemic gaps, strategic collaboration among key stakeholders, including government, healthcare providers, private health insurance and non-governmental organisations, is needed. To overcome the inaccessibility to healthcare, priorities should include improving rural infrastructure, expanding healthcare workforce distribution and oncology capacity.

While EBV-based screening approaches, including VCA/EBNA1-IgA serology and plasma EBV DNA quantification, have shown promise in improving early detection and reducing NPC-specific mortality, concerns remain regarding low positive predictive value and variability in laboratory performance. Consequently, population-wide screening of NPC has never been conducted in SEA countries. Promisingly, new biomarkers such as EBV serological marker anti-BNLF2b total antibody (P85-Ab) and sequencing-based plasma EBV DNA fragment-size profiling have shown encouraging potential for screening of NPC [116, 117]. Large-scale prospective efforts in China are currently evaluating their utility. In SEA where many countries are classified as low- and middle-income, screening strategies must rely on simple, affordable, and scalable tools. Such tools must be cost-effective and can be readily adopted by existing laboratories in these countries to ensure that the approaches are feasible beyond tertiary centres and scalable at the population level. Continued research into innovative biomarkers remains essential.

Artificial intelligence (AI) has the power to enhance every stage of cancer management from screening, early detection, treatment planning prognostication and post-treatment surveillance. The development of AI tools for the management of NPC is a rapidly evolving field [118] and a recent systematic review indicated that the focus of attention has been in the areas of diagnosis, prognosis and auto-contouring/radiotherapy planning [119]. Digital pathology is now being used for primary cancer diagnosis and AI has the potential to augment this approach in many cancer types, including NPC. For example, a deep-learning model was described that can distinguish NPC from nasopharyngeal inflammation and lymphoid hyperplasia, using whole slide H&E images [120]. Additionally, a recent study reported the development of a multimodal deep-learning-based digital score that incorporated data from H&E stained slides and clinical information that predicted distant metastasis and survival [121]. The integration of digital pathology and AI has the potential to improve clinical outcomes. In the context of the challenges of NPC in much of SEA, the immediate impact of AI is likely to be in the area of screening/early detection, particularly for high-risk populations and those living in remote areas. In this regard, two recent studies using AI together with endoscopic images show great potential. Shi and co-workers describe the development of an AI-assisted strategy to improve diagnostic accuracy in a clinical setting [122]. In addition, a deep learning-based smartphone app using endoscopic images has been developed for the early detection of NPC [123]. The app termed “Nose-Keeper,” was reported to demonstrate high accuracy with 96% sensitivity and 99.9% specificity [123]. Such resources and approaches are likely to prove particularly valuable in many communities across SEA in the coming years.

Given the substantial burden of NPC across SEA, characterised by late-stage presentation and pronounced socioeconomic disparities, there is an urgent need for coordinated regional action. Strengthening healthcare infrastructure, expanding early detection efforts through community education and healthcare provider training, and developing affordable screening strategies are critical priorities. Addressing these gaps holistically is essential to reduce delays in diagnosis and treatment, improve survival, and narrow the inequality in NPC outcomes across SEA.
